# Phase separation promotes the activity of HECT E3 ligases—A word of caution

**DOI:** 10.1073/pnas.2402551121

**Published:** 2024-03-25

**Authors:** Daniela Eichbichler, Christine Bernecker, Florian Stengel, Martin Scheffner

**Affiliations:** ^a^Department of Biology, University of Konstanz, 78457 Konstanz, Germany

Liquid–liquid phase separation (LLPS) has been recognized as a fundamental process in the organization of membrane-less reaction compartments in cells (1, 2). At the heart of LLPS are multivalent protein–protein and protein–RNA interactions that can be modulated by posttranslational modifications (3). There is increasing evidence that modification of proteins by ubiquitin (“ubiquitination”) and in particular by ubiquitin chains (“polyubiquitination”) plays an important role in LLPS (4). In a simplified view, ubiquitin chains come in many different linkage types (5), display high conformational plasticity, and due to their polymeric nature can act as multivalent interaction platforms prone to LLPS.

Ubiquitination is mediated by the consecutive action of E1 ubiquitin-activating enzymes, E2 ubiquitin-conjugating enzymes, and E3 ubiquitin–protein ligases (6). Besides their critical role in substrate recognition, E3s are characterized by their propensity to ubiquitinate themselves (auto-ubiquitination). Recently, Li et al. (7) reported that auto-polyubiquitination of HECT E3 family members not only induces LLPS, but in turn, LLPS promotes the ligase activity of HECT E3s. The latter finding is of particular interest, as it would provide a new regulatory mechanism for HECT E3s. The conclusion that LLPS stimulates HECT E3 activity is based on two lines of evidence. First, Li et al. studied a fusion protein consisting of the low complexity domain (LCD) of FUS, which is known to induce LLPS (8), and the catalytic HECT domain of Itch. In comparison to the HECT domain alone, the fusion protein was more active in auto-ubiquitination assays, and this correlated with the propensity for LLPS. While this is in line with the notion that LLPS promotes E3 activity, it does not prove it (as it cannot be excluded that fusion of an unrelated protein, i.e., LCD, has an intrinsic effect on the catalytic properties of the HECT domain, e.g., by affecting its conformational dynamics). Second, and more importantly, 1,6-hexanediol (HD), which is widely used in LLPS studies as it interferes with LLPS (9), inhibited auto-polyubiquitination of HECT E3s, while the effect of 2,5-HD, which supposedly does not affect HECT E3-induced LLPS (respective data were not provided by Li et al.), on HECT E3 auto-polyubiquitination was less significant.

**Fig. 1.**
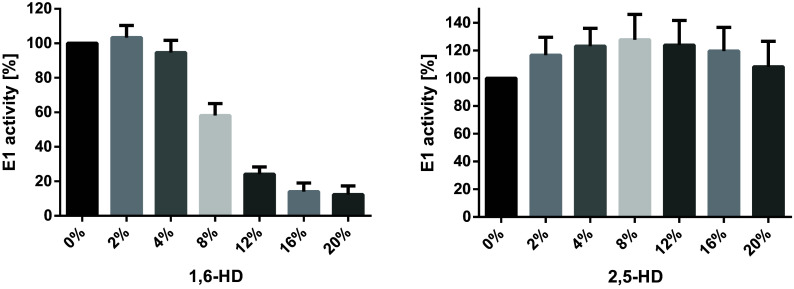
1,6-HD inhibits E1 activity. Recombinant E1 (UBA1) was incubated with ubiquitin in the absence or presence of increasing concentrations of 1,6-HD or 2,5-HD as indicated at 37 °C for 30 min in the presence of pyrophosphatase (New England Biolabs, M2403L). E1 activity (i.e., ATP hydrolysis) was determined by the Malachite Green assay (Sigma-Aldrich, MAK307-1KT), normalized to the reaction in the absence of the respective HD (set to 100%), and the mean and SD were plotted using GraphPad Prism8 (n = 6).

At first glance, the data obtained with the HD isomers seem to provide compelling evidence that LLPS stimulates the activity of HECT E3s. However, as auto-ubiquitination involves three different enzymes, an alternative explanation for the inhibitory effect of 1,6-HD is that it negatively affects the activity of the E1 and/or E2 enzymes rather than HECT E3s. To address this possibility, we determined the effect of the HD isomers on E1 activity by measuring ATP hydrolysis, which is essential for ubiquitin activation and thus ubiquitination (10). This showed that 1,6-HD strongly interferes with E1 activity in a dose-dependent manner, while 2,5-HD does not (Fig. 1). Thus, we propose that i) the negative effect of 1,6-HD on HECT E3 auto-polyubiquitination is not due to its potential to interfere with LLPS, and ii) additional experiments/evidence are required to prove that LLPS stimulates the activity of HECT E3s.
